# The complete chloroplast genome sequence of *Goodyera schlechtendaliana* in Korea (Orchidaceae)

**DOI:** 10.1080/23802359.2019.1641439

**Published:** 2019-07-24

**Authors:** Sang-Hun Oh, Hwa Jung Suh, Jongsun Park, Yongsung Kim, Sangtae Kim

**Affiliations:** a Department of Biology, Daejeon University, Daejeon, Korea;; b Department of Biology, Sungshin University, Seoul, Korea;; c Infoboss Co., Ltd., Seoul, Korea;; d InfoBoss Research Center, Seoul, Korea

**Keywords:** *Goodyera*, chloroplast genome, *Goodyera schlechtendaliana*, Korea, Orchidaceae

## Abstract

*Goodyera schlechtendaliana* is a common orchid species in East Asia, providing a case to study phylogeographic structure of understory plants in warm temperate forests. Here, we present the complete chloroplast genome of the Korean *G. schlechtendaliana*. Its length is 153,801 bp and it has four subregions; 82,683 bp of large-single-copy and 18,048 bp of small-single-copy regions are separated by 26,535 bp of inverted repeat regions, including 133 genes (86 protein-coding genes, eight rRNAs, and 39 tRNAs). Phylogenetic analyses suggest that the chloroplast genomic data should be useful in future phylogeographic and phylogenetic studies of *Goodyera*.

*Goodyera schlechtendaliana* Rchb. f. (Orchidaceae) is a common orchid widely distributed in the Himalayas, Sumatra, China, Taiwan, Korea, and Japan, occupying shady places with moist and well-drained soils. It is characterized by having creeping rhizomes, white variegated markings on the adaxial surfaces of the leaves, saccate labellum, two sectile pollinia attached to a viscidium, and a single stigmatic lobe in Orchidaceae (Chen et al. 2009; Hu et al. 2016). It is often cultivated as an ornamental because of the patterns on the leaves. Both sexual and clonal reproduction occur in *G. schlechtendaliana* (Brzosko et al. 2013). Phylogeographic structure representing differentiation among populations has not been studied despite the wide distribution range of the species. Chloroplast genome is useful to trace the seed movement and infer the geographic structure.

The complete chloroplast genome of *G*. *schlechtendaliana* from southern part of Korea (34°41′23.28″N, 125°11′48.49″E) was determined to be used in understanding of infraspecific variation. Total DNA was extracted from fresh leaves collected on Hongdo Island in Shinan-gun, Jeollanam-do, Korea (voucher in the herbarium of Daejeon University (TUT); *Oh 7171*) using a DNeasy Plant Mini Kit (QIAGEN, Hilden, Germany). Paired-end sequencing was performed using HiSeq4000 (Illumina, San Diego, USA) of Macrogen Inc., Korea. *De novo* assembly was performed using Velvet 1.2.10 (Zerbino and Birney 2008), and gap sequences were filled by SOAPGapCloser 1.12 (Zhao et al. 2011), BWA 0.7.17 (Li 2013), and SAMtools 1.9 (Li et al. 2009). Geneious R11 11.0.5 (Biomatters Ltd., Auckland, New Zealand) was used for genome annotation based on *G. schlechtendaliana* chloroplast genome (MK134679; Oh et al. 2019).

The chloroplast genome of Korean *G. schlechtendaliana* (GenBank accession: MK144665) is 153,801 bp (the GC-ratio is 37.2%) and has four subregions: 82,683 bp of large-single-copy (GC-ratio, 34.9%) and 18,048 bp of small-single-copy (GC-ratio, 29.7%) regions are separated by 26,535 bp each of inverted repeats (IR; GC-ratio, 43.3%). It contains 133 genes (86 protein-coding genes, eight rRNAs, and 39 tRNAs) with 19 genes (seven protein-coding genes, four rRNAs, and eight tRNAs) duplicated in the IR regions.

Twelve complete chloroplast genomes, includig eight from four species of *Goodyera*, two from closely allied groups, and two outgroups, were aligned using MAFFT 7.388 (Katoh and Standley 2013). Phylogenetic trees were constructed using the neighbor-joining (with 10,000 bootstrap repeats) and maximum likelihood methods (with 1000 bootstrap repeats) in MEGA X (Kumar et al. 2018).

The phylogenetic tree shows that *G. schlechtendaliana* from Korea forms strongly supported clade with other accessions of *G*. *schlechtendaliana* from China (Figure 1A). The result agrees with morphology and previous phylogenetic analysis based on nuclear ITS regions (Hu et al. 2016). Comparison of five chloroplast genomes of *G*. *schlechtendaliana* showed 200–844 single nucleotide polymorphisms and 414–2133 insertions and deletions among accessions (Figure 1B), suggesting a high level of infraspecific variation compared with those in *Pseudostellaria* (Kim et al. 2019) and *Coffea* (Park et al. 2019). The chloroplast genome will be a useful resource for investigation of phylogeographic structure within *G*. *schlechtendaliana* and for understanding phylogenetic relationship of *Goodyera*.

**Figure 1.**
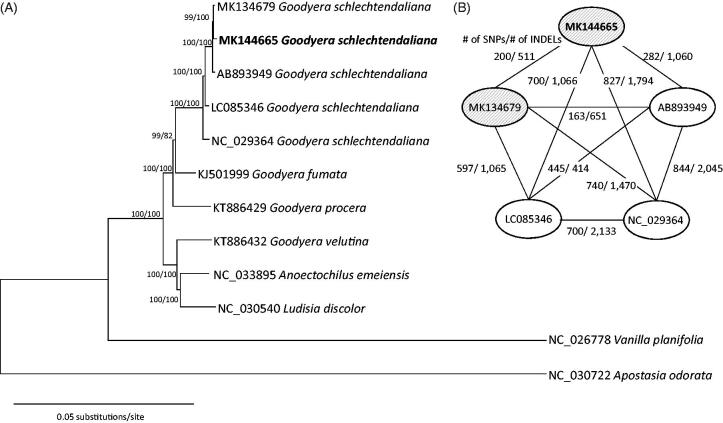
(A) A maximum-likelihood tree using chloroplast genomes of *G. schlechtendaliana* from Korea (MK144665 in this study and MK134679) and previously published related taxa: *G. schlechtendaliana* from China (AB892949, LC085346, and NC_029364), *G. fumata* (KJ501999), *G. procera* (KT886429), *G. velutina* (KT886432), *Ludisia discolour* (NC_030540), *Anoectochilus emeiensis* (NC_033895), and two outgroup species, *Vanila planifolia* (NC_026778) and *Apostasia odorata* (NC_030722). Bootstrap values using the neighbor-joining and maximum-likelihood methods are indicated above the branch. (B) Pairwise comparisons of five chloroplast genomes of *G. schlechtendaliana*. Numbers of single nucleotide polymorphisms (SNPs) and insertions and deletions (INDELs) between each pair are indicate on the branch. Filled eclipses indicate *G. schlechtendaliana* originated from Korea and opened eclipses mean *G. schlechtendaliana* originated from China.
